# Path Planning of Quadrupedal Robot Based on Improved RRT-Connect Algorithm

**DOI:** 10.3390/s25082558

**Published:** 2025-04-18

**Authors:** Xiaohua Xu, Peibo Li, Jiangwu Zhou, Wenzhuo Deng

**Affiliations:** College of Mechanical Engineering, Donghua University, Shanghai 201620, China; 2221136@mail.dhu.edu.cn (X.X.); 2221246@mail.dhu.edu.cn (J.Z.); 2221186@mail.dhu.edu.cn (W.D.)

**Keywords:** quadrupedal robot, rectangular limit sampling, adaptive dynamic step size, reverse greedy algorithm, path smoothing

## Abstract

In view of the large randomness, redundant path nodes, and low search efficiency of RRT-connect in a complex obstacle environment, this study intends to develop a path-planning method combining RRT-connect and Informed RRT*. First, to solve the problem of large sampling randomness, the Informed RRT* algorithm is combined to adopt a simpler rectangle and limit the sampling range to the rectangle. Second, for the poor quality of the search path, the dynamic step size is used for growth extension, the reverse greedy algorithm is used to delete redundant nodes, the spline curve is used to smooth the path such that the position meets the cubic spline curve and the speed meets the quadratic spline curve, and the final path is optimized. Finally, the proposed algorithm is verified in the simulation and real world using a self-developed quadrupedal robot. Compared with the original RRT-connect algorithm, the first solution time, total number of nodes, and initial path cost were reduced by more than 11%, 8.5%, and 2.5%, respectively.

## 1. Introduction

Quadrupedal robots have become the focus of mobile robot research owing to their excellent terrain adaptability and flexible control ability. To better cope with complex terrain, quadrupedal robots need to have the ability of path planning, which is the key to realizing autonomous navigation in complex environments. Common robot path planning methods are divided into three categories. First, search-based path planning, such as Dijkstra [1], A* [2], and the JPS algorithm [3]. Path planning is based on intelligent algorithms including reinforcement learning [4], deep learning [5], genetic algorithms [6], and ant colony algorithms [7]. In addition, sampling-based path planning methods including Probabilistic Roadmaps (PRM) [8] and Rapidly Exploring Random Trees (RRT) have been widely adopted. The sampling-based algorithm can deal with complex path planning problems in multi-dimensional space, and is suitable for quadruped robots and robotic arms, etc., with the advantages of fast convergence, small computation, and easy implementation. It has attracted much attention, and many variants have been derived. However, its disadvantages are also obvious; that is, such algorithms are usually not optimal, and only when the planning time is sufficiently long and the sampling times are sufficient can an effective path be found.

To address these challenges, researchers globally have proposed various enhancements. Building upon the original RRT framework, Kuffner [9] and colleagues developed the RRT-connect variant that accelerates convergence through simultaneous search tree construction from both start and goal configurations. Karaman and Frazzoli’s [10] RRT* algorithm significantly advanced the field by introducing parent node reselection and rewiring mechanisms, thereby achieving asymptotic optimality through iterative path optimization. Building on these developments, Klemm’s [11] RRT*-connect algorithm successfully hybridized the advantages of RRT-connect and RRT*, preserving the asymptotic optimality while enhancing convergence rates. Parallelly, Gammell’s [12] research team advanced the Informed RRT methodology, which implements solution-space biasing after initial path discovery to systematically refine path quality. However, Informed RRT* has problems with other one-way tree planners, especially when the target configuration is hidden behind a narrow channel and takes time to reach the target configuration. Building upon this foundation, Mashayekhi [13] and collaborators developed the Informed RRT-connect algorithm, which employs state-space optimization screening to simultaneously reduce both path costs and computational iterations. Norby and Johnson [14] proposed a motion-planning algorithm for a legged robot that can build a long-vision dynamic programming in real time. The algorithm processes motion primitives with attitude and flight phases through a reduced-order dynamic model, and supports the RRT-connect framework for rapid exploration. Although the algorithm planner is fast, there is still room for improvement. Kang [15] proposed a reconnection method based on triangular inequalities for RRT-connect algorithms. Compared to the RRT algorithm, this method ensures that the planning time is closer to the optimal value. Evaluation results reveal significant improvements over conventional RRT, with measured reductions in both planning duration and trajectory distance. The enhanced efficiency persists even when matching the computational resources of RRT-connect implementations. Zhang [16] et al. proposed an enhanced RRT-connect algorithm to overcome the poor sampling uniformity and low planning efficiency of conventional RRT-connect methods. The proposed approach implements a two-stage optimization process. First, it integrates both goal-biased and explored-node-biased sampling strategies to significantly improve point distribution quality. Then, it introduces an advanced node evaluation scheme that dynamically selects optimal parent nodes within defined search regions, enabling concurrent optimization of sampling nodes and local path segments while effectively minimizing total path planning costs. Zheng [17] et al. proposed an improved method integrating greedy search strategy and cubic Bezier curve optimization to address the shortcomings of conventional RRT-connect algorithms in static environments, including inadequate obstacle avoidance reliability, low planning efficiency, and unsatisfactory path smoothness. The enhanced approach features a two-phase optimization process: first employing greedy search for rapid feasible solution acquisition, followed by cubic Bezier curve-based path smoothing, thereby significantly improving both path quality and planning efficiency while maintaining robust obstacle avoidance capability. Luo et al. [18] proposed an enhanced RRT-connect and DWA fusion algorithm that improves path planning performance through key technical innovations. The method optimizes sampling strategies and collision detection for global planning while employing fuzzy logic to dynamically adjust local navigation parameters. Experimental validation confirms its effectiveness in complex environments, demonstrating both real-time capability and reliable obstacle avoidance. Li [19] et al. proposed an improved RRT-connect algorithm with target-oriented sampling and path post-processing for pruning robot navigation in orchard environments. By incorporating target guidance strategy and trajectory smoothing techniques, the enhanced algorithm demonstrates superior path planning performance compared to conventional methods. Patel [20] et al. proposed an innovative path planning algorithm that integrates probabilistic RRT (pRRT) with RRT-connect techniques. This approach employs a novel multi-tree cooperative growth strategy, where intermediate waypoints are strategically selected around obstacles to construct multiple subtrees connecting the start, goal, and intermediate points, ultimately forming a complete collision-free path.

This study proposes an improved RRT-connect algorithm designed to address three persistent challenges in sampling-based path planning: large sampling randomness, low search efficiency, and poor search-path quality. First, for the problems of large sampling randomness and low search efficiency, the Informed RRT* algorithm is combined with a more concise rectangle to limit the sampling range. Second, to solve the problem of poor quality of the search path, a dynamic step size is used for growth extension, the reverse greedy algorithm is used to delete redundant nodes, and the cubic spline curve is used to smooth the path, so as to optimize the final path. Comprehensive testing on a custom robotic platform establishes measurable advances in operational efficiency relative to existing approaches, with consistent performance gains observed in both digital and physical testbeds.

## 2. Improved RRT-Connect Algorithm

Compared with the RRT-connect algorithm, although its planning efficiency has improved, there are still some notable issues, such as excessive randomness, insufficient scalability, numerous inflection points in the path, and a large number of redundant nodes. These problems not only affect the overall performance of the algorithm but also limit its widespread use in practical applications. This study presents a comprehensive enhancement framework for the RRT-connect algorithm, delivering four key innovations: (1) integration of Informed-RRT* with rectangular sampling to focus exploration while maintaining goal-directed behavior; (2) implementation of an adaptive step-size mechanism that dynamically responds to environmental complexity, optimizing node generation efficiency; (3) development of a reverse greedy optimization technique for path refinement, eliminating unnecessary waypoints while minimizing trajectory length; and (4) incorporation of cubic spline smoothing to ensure motion continuity and executability. Collectively, these advancements substantially improve planning speed, solution quality, and real-world deployment capabilities of the path planning system.

### 2.1. Integration of Rectangle-Based Informed-RRT* Algorithm

Compared with the RRT-connect algorithm, which has already demonstrated improved planning efficiency, several significant challenges remain that hinder its performance in practical applications. The algorithm exhibits several limitations: uncontrolled stochasticity in node sampling, constrained environmental adaptability, suboptimal path smoothness with excessive curvature variations, and inefficient node utilization patterns, all of which degrade computational performance and solution quality. To address these limitations and further enhance the performance of the RRT-connect algorithm, this study proposed a comprehensive set of improvements across four key areas. First, the Informed-RRT* algorithm, based on a rectangular sampling region, is introduced to focus on the search space and improve the algorithm’s ability to converge toward the goal. Second, a dynamic step-size mechanism is implemented to adaptively adjust the step length based on environmental complexity, thereby reducing redundant nodes and improving search efficiency. Third, a reverse greedy combination algorithm is developed to optimize the trajectory by minimizing unnecessary inflection points and shortening the overall path length. Finally, cubic spline curve smoothing techniques were applied to refine the path, ensuring smoother and more continuous trajectories that are better suited for real-world applications. These methodological refinements collectively enhance the RRT-connect framework’s core capabilities, optimizing planning speed, trajectory quality, and environmental versatility across heterogeneous operating conditions.

The Informed-RRT* methodology utilizes elliptical sampling domains rather than global uniform sampling, effectively eliminating the problem of superfluous branch generation characteristic of conventional RRT* implementations while enhancing both exploration efficiency and convergence rates. However, elliptical calculations are relatively complex. The proposed method implements a rectangular sampling strategy, where the boundary coordinates *P*_1_, *P*_2_, *P*_3_, and *P*_4_ are derived geometrically from the initial point *P_init_* and target point *P_goal_* configurations as illustrated, and the coordinates of points *P*_1_, *P*_2_, *P*_3_, and *P*_4_ are denoted as *P*_1_(*x*_1_,*y*_1_), *P*_2_(*x*_2_,*y*_2_), *P*_3_(*x*_3_,*y*_3_), and *P*_4_(*x*_4_,*y*_4_) and the rectangular region is determined based on these four points. The sampling was performed within this rectangle.

Let the coordinates of the starting point *P_init_* be (*x_init_*,*y_init_*) and the coordinates of the target point *P_goal_* be (*x_goal_*,*y_goal_*). The length of the rectangle is

(1) $${long}_{1}=\sqrt{{\left(x_{goal}-x_{init}\;\right)}^{2}+{\left(y_{goal}-y_{init}\;\right)}^{2}},$$

The rectangular domain’s width is computed as a fixed proportion of its longitudinal dimension, specifically

(2) $${wide}_{1}=0.5*{long}_{1},$$

The coordinates of the center point of the rectangle are $\left(\frac{x_{goal}-x_{init}}{2},\frac{y_{goal}-y_{init}}{2}\right)$.

The corner α can be obtained using the following formula:

(3) $$\alpha =\tan^{-1}\left(\frac{y_{goal}-y_{init}}{x_{goal}-x_{init}}\right),$$

The coordinates of $P_{1}$($x_{1},y_{1}$), $P_{2}$($x_{2},y_{2}$), $P_{3}$($x_{3},y_{3}$), and $P_{4}$($x_{4},y_{4}$) are

(4) $$P_{1}(x_{goal}-\frac{wide}{2}*\sin\alpha ,y_{goal}+\frac{wide}{2}*\cos\alpha \;),$$

(5) $$P_{2}(x_{goal}+\frac{wide}{2}*\sin\alpha ,y_{goal}-\frac{wide}{2}*\cos\alpha \;),$$

(6) $$P_{3}(x_{init}-\frac{wide}{2}*\sin\alpha ,y_{init}+\frac{wide}{2}*\cos\alpha \;),$$

(7) $$P_{4}(x_{init}+\frac{wide}{2}*\sin\alpha ,y_{init}-\frac{wide}{2}*\cos\alpha \;),$$

**Figure 1:**
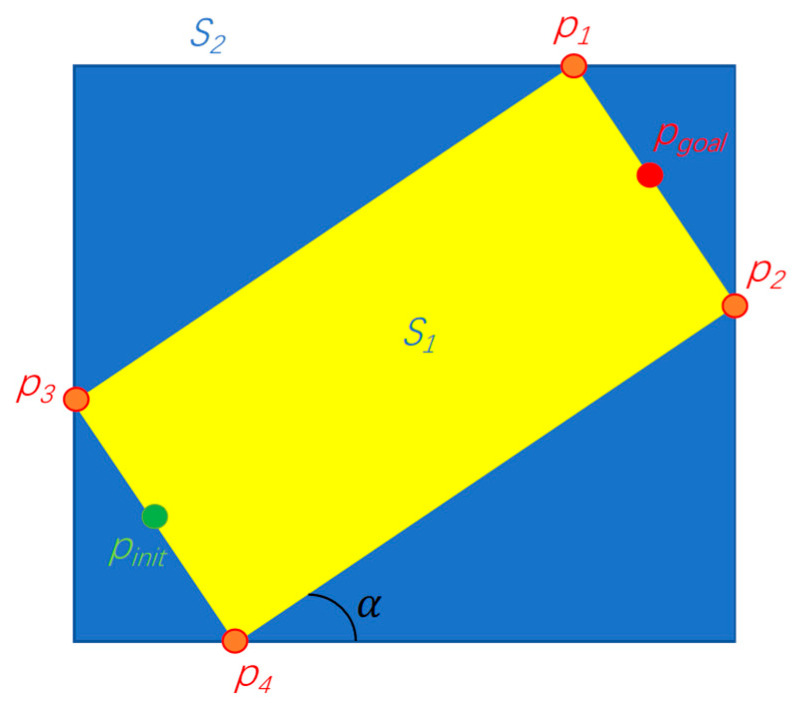
Sampling area.

The rectangular region *S*_2_ is determined by the four points *P*_1_, *P*_2_, *P*_3_, and *P*_4_:

(8) xmax=max(x1,x2,x3,x4)xmin=min(x1,x2,x3,x4)ymax=max(y1,y2,y3,y4)ymin=min(y1,y2,y3,y4),

The region *S*_2_ is restricted in Formula (9):

(9) x<xmaxx>xminy<ymaxy>ymin,

The sampling area *S*_2_ is shown in Figure 1.

### 2.2. Dynamic Step Size Exploration

**Figure 2:**
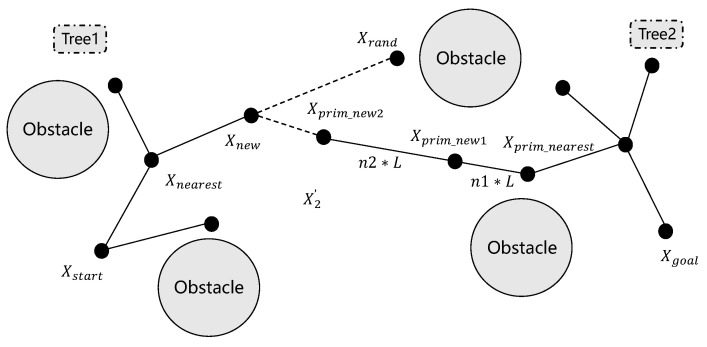
Dynamic step growth diagram.

The bidirectional growth mechanism in RRT-connect inherently produces substantial node redundancy, creating unnecessary computational burden and compromising algorithmic efficiency. Furthermore, the method demonstrates limited global search capability, often terminating in locally optimal solutions when confronted with dense obstacle configurations. To address these issues, this study proposes replacing the traditional fixed step size with a dynamic step size mechanism for tree growth and extension. The adaptive step-size mechanism dynamically responds to both environmental complexity and inter-tree distance, optimizing exploration efficiency while minimizing redundant node generation. This spatial awareness concurrently prevents local optima entrapment through systematic search space coverage. The dynamic step growth process is illustrated in Figure 2, which demonstrates how the step size varies during the tree expansion process, enabling smoother and more efficient convergence of the two trees. The dynamic step-size strategy significantly enhances RRT-connect’s operational robustness and path-planning efficacy through adaptive exploration control.

As illustrated in the figure, when Tree1 generates a new node, denoted as node $x_{new}$, Tree2 identifies the parent node that is closest to node $x_{new}$ based on the Euclidean distance metric. Tree2’s nearest ancestral node, designated $x_{prim\_new}$, is determined through Euclidean distance minimization. Subsequently, the connection direction between the parent node $x_{prim\_new}$ and the randomly generated point determines the direction in which Tree2 expands. The expansion of Tree2 is then executed with a dynamic step size, referred to as step size $L^{*}$. This dynamic step size is crucial for controlling the growth of Tree2 and is calculated using a specific formula that considers various factors to ensure optimal expansion. The dynamic step size $L^{*}$ is computed through real-time assessment of both tree topology and environmental constraints, enabling optimal balance between exploration efficiency and target-directed expansion. This self-adjusting mechanism continuously optimizes tree growth patterns, significantly improving convergence behavior and computational performance.

(10) $$L^{*}=N*L$$

Here, *L* is the set step-size and *N* is the dynamic coefficient, which is determined by whether the space constraint is satisfied. *L* is a critical parameter governing the expansion efficiency of the RRT-connect trees. *L* represents the maximum local search radius during tree expansion, directly tied to the robot’s kinematic constraints:

(11) $$L=v_{max}*∆t+\epsilon$$

where $v_{max}$ is the velocity upper bound, experimentally configured as 1.5 m/s based on our robotic platform’s dynamic performance thresholds; the control cycle time ∆t is set to 0.03 s; and ϵ is the safety margin that is set to 0.05 m to avoid collisions.

*N* is given by

(12) $$N=\left\{\begin{array}{c} 1\;if\;\mathrm{not}\;CON(x_{prim\_new},x_{prim\_nearest})\\ N+1\;\mathrm{other} \end{array}\right.,$$

### 2.3. Reverse Greed Algorithm

**Figure 3:**
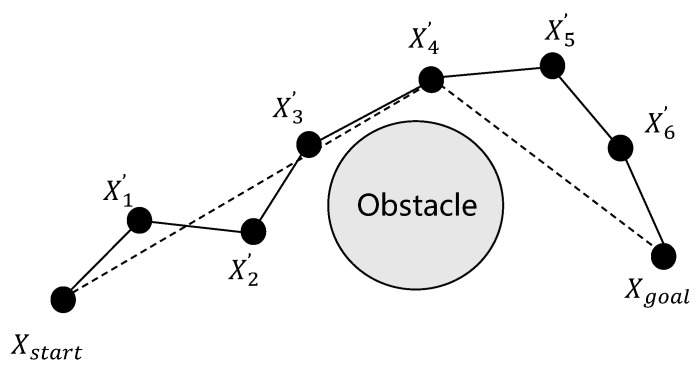
Reverse greed algorithm.

The inverse greedy algorithm is employed to optimize the fragmented path, which often contains unnecessary nodes that can be eliminated to create a more efficient and streamlined trajectory. The path optimization results following node elimination are illustrated in Figure 3, demonstrating the following computational procedure. Commencing from the target configuration $x_{goal}$, the algorithm iteratively establishes connections with preceding trajectory node $x_{n}^{\prime }$. It then checks whether there is an obstacle between $x_{goal}$ and the current node $x_{n}^{\prime }$. If no obstacle is detected, then all intermediate nodes between these two points are deleted, thereby simplifying the path. However, if an obstacle is found between node $x_{goal}$ and node $x_{n}^{\prime }$, the algorithm repeats the same steps from node $x_{n+1}^{\prime }$, continuing to evaluate and remove unnecessary nodes until it has traversed the entire path and reached the starting point $x_{start}$. This iterative process ensures that the final path is both obstacle-free and optimized for efficiency, thereby reducing the number of nodes while maintaining the feasibility and smoothness of the trajectory. The proposed optimization method enhances path quality substantially, particularly for real-world deployment scenarios demanding computationally efficient and geometrically simplified trajectories.

### 2.4. Trajectory Optimization and Smoothing Processing

The path generated by the aforementioned method consists solely of sampled nodes that must satisfy both static and dynamic constraints to ensure feasibility and practicality in real-world applications. The robot’s operational constraints encompass both static and dynamic requirements. Static constraints derive from the platform’s physical morphology, including center-of-mass elevation and limb dimensions, ensuring stable locomotion and obstacle avoidance through mathematically enforced boundaries. Dynamic constraints govern motion characteristics such as velocity profiles, acceleration thresholds, and torque outputs, guaranteeing kinematically viable and energy-efficient operation. In particular, the static constraints are explicitly formulated in Equation (13), which provides a detailed mathematical representation of how the height of the center of mass and the leg height influence the robot’s movement. The integration of these kinematic and dynamic constraints into the path-planning framework guarantees the generation of both obstacle-avoidant and dynamically executable trajectories. This constraint-aware planning approach facilitates reliable navigation through complex terrains while preserving locomotor stability and operational efficiency.

(13) $$\left\{\begin{array}{cc} h_{center}\geq h_{min} & \forall t\;\in \left[0,t_{1}+t_{2}\right]\\ h_{leg}\leq h_{max} & \forall t\;\in \left[0,t_{1}\right] \end{array}\right.,$$

Here, $h_{center}$ is the height of the center of mass, $h_{leg}$ is the height of the leg, $h_{min}$ and $h_{max}$ are the clearance threshold, $t_{1}$ is the support phase time, and $t_{2}$ is the flight phase time.

The system’s dynamic operational requirements further incorporate three critical mechanical constraints: ground reaction forces, actuator torque limits, and frictional boundary conditions. These are mathematically defined as

(14) |fGRF|≤fmaxf3≥0|τ|≤τmaxμf3≥f12+f22,

where $f_{GRF}$ is the ground reaction force, $f_{max}$ is the fixed maximum ground reaction force, τ is the torque, $\tau_{max}$ is the maximum torque threshold, $f_{3}$ is the normal component of the ground reaction force, $f_{1}$ and $f_{2}$ are the tangential components of the ground reaction force, and μ is the friction coefficient. Within the limits of these constraints, the ground reaction force vector and torque at touchdown and takeoff are randomly sampled and rotated into the world coordinate system.

**Figure 4:**
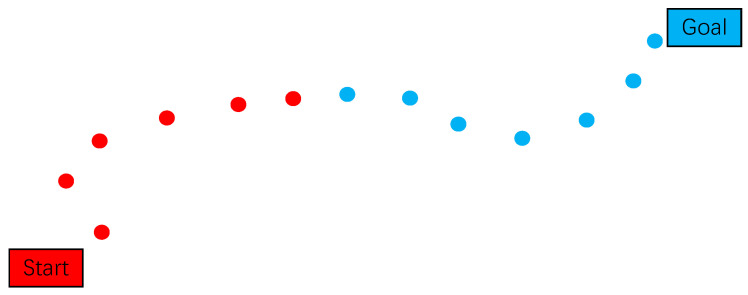
Schematic diagram of optimized RRT-connect algorithm.

Node interconnection is strictly governed by a comprehensive set of kinematic and dynamic constraints, including but not limited to collision avoidance, torque limitations, and energy consumption thresholds. The resulting trajectory, as visualized in Figure 4, demonstrates the characteristic bidirectional exploration pattern: the red trajectory segments (left) represent nodes from the initial search tree rooted at the starting configuration, while the blue segments (right) denote nodes from the goal-oriented search tree. The algorithmic process implements a synchronized bidirectional expansion mechanism, where both subtrees progressively extend toward each other through iterative sampling and validation cycles. This systematic exploration continues until meeting the convergence criterion, typically defined by configurational proximity threshold or maximum iteration count, at which stage the algorithm successfully constructs a continuous, constraint-satisfying path through concatenation of the respective tree segments.

Trajectory smoothing is achieved through application of cubic spline interpolation, ensuring continuous curvature profiles along the optimized path. To ensure that the smoothed trajectory satisfies the spatial state, it is necessary to generate adjacent nodes around path points. We take nodes $x_{n-1}$ and $x_{n+1}$ before and after path point $x_{n}$ as directions and step as steps to generate a new adjacent node.

Relying solely on the scattered nodes generated by the aforementioned method is clearly insufficient, because it does not provide the level of detail required for precise and smooth motion planning. To address this, further refinement of the path is necessary, which involves performing denser node interpolation between the existing nodes. This interpolation process ensures that the path is continuous and finely detailed, thereby enabling the robot to follow it accurately. Additionally, the minimum interval time for planning is set to 0.03 s, ensuring that the trajectory is both temporally and spatially optimized for real-time execution.

The interpolation procedure is rigorously constrained by kinematic and dynamic requirements, guaranteeing both trajectory feasibility and continuity. For instance, the velocity profile is determined using quadratic spline curve interpolation, which ensures smooth transitions between nodes and avoids abrupt changes in the speed. The position trajectory is calculated using cubic spline curve interpolation, which provides a higher degree of continuity and smoothness, making it suitable for complex motion planning. The use of a cubic spline curve for the position trajectory allows it to be mathematically expressed as Equation (15). By incorporating these interpolation techniques and adhering to the specified constraints, the proposed method ensures that the generated path is not only precise but also dynamically feasible, enabling the robot to execute smooth and efficient movements in real-world scenarios.

(15) $$q(t)=a_{0}+a_{1}t+a_{2}t^{2}+{a_{3}t}^{3}$$

Here,  q(t) is a function of the position trajectory and $a_{0}$, $a_{1}$, $a_{2}$, and $a_{3}$ are unknown parameter sets.

Find the first and second derivative, respectively:

(16) $$q{\left(t\right)}^{\prime }=a_{1}+2a_{2}t+{3a}_{3}t^{2},$$

(17) $$q{\left(t\right)}^{\prime \prime }=2a_{2}+6a_{3}t,$$

where $q{\left(t\right)}^{\prime }$ is the velocity function and $q{\left(t\right)}^{\prime \prime }$ is the acceleration function.

According to the initial conditions, Equation (18) can be obtained using the above equation. The calculation formula for the support phase is given by Equation (18):

(18) $$\left\{\begin{array}{c} \ddot{q}(t)=({\ddot{q}}_{TF}-{\ddot{q}}_{TG})\frac{t}{t_{1}}+{\ddot{q}}_{TG}\\ \dot{q}(t)=({\ddot{q}}_{TF}-{\ddot{q}}_{TG})\frac{t^{2}}{{2t}_{1}}+{\ddot{q}}_{TG}t+{\dot{q}}_{TG}\\ q(t)=({\ddot{q}}_{TF}-{\ddot{q}}_{TG})\frac{t^{3}}{6t_{1}}+{\ddot{q}}_{TG}\frac{t^{2}}{2}+{\dot{q}}_{TG}+q_{TG} \end{array}\right.,$$

where TG is the moment of touching the ground, that is, the beginning of the standing stage, and TF is the moment of jumping, that is, the end of the standing stage.

During the flight phase, the system’s motion is governed solely by gravitational acceleration, which produces the parabolic trajectory described in Equation (19):

(19) $$\left\{\begin{array}{c} \ddot{q}(t)=g\\ \dot{q}(t)=g(t-t_{1})+{\dot{q}}_{TO}\\ q(t)=\frac{g{\left(t-t_{1}\right)}^{2}}{2}+{\dot{q}}_{TO}(t-t_{1})+q_{TO} \end{array}\right.,$$

**Figure 5:**
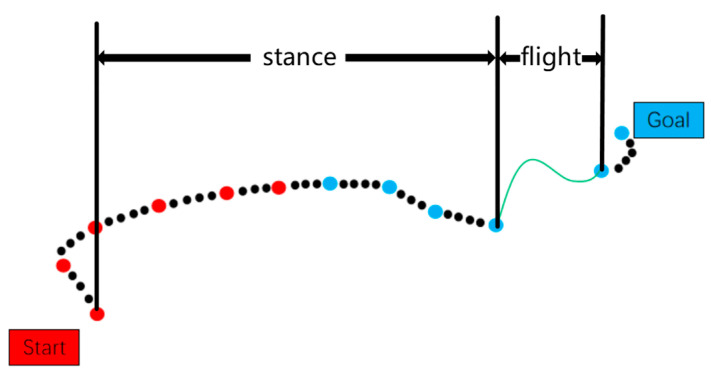
Node interpolation diagram.

Figure 5 shows the schematic diagram of nodal interpolation during the path planning process.

**Figure 6:**
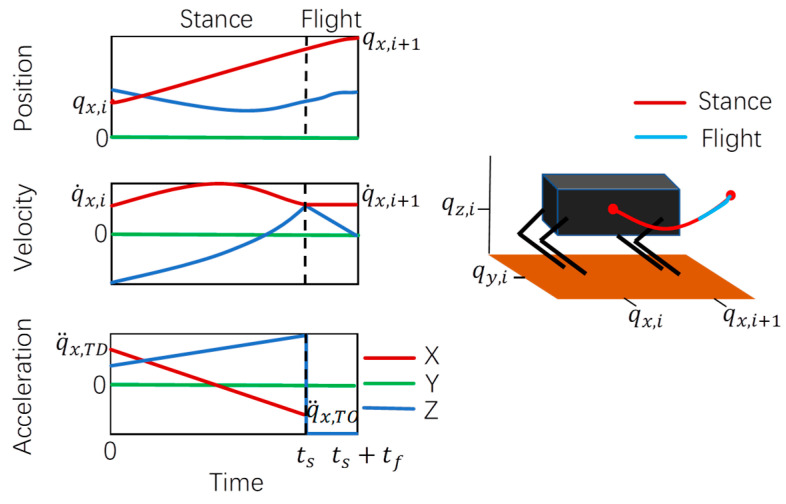
Robot velocity, acceleration, displacement, and time relationship diagram.

The relationship between velocity, acceleration, displacement, and time of Equations (18) and (19) is drawn, as shown in Figure 6.

The displacement of these nodes enables velocity-based motion control of the robot, while acceleration governs the transition between support and flight phases. In addition, the attitude and angular velocity of the robot must be calculated, which are the input variables when NMPC is used for trajectory tracking. The proposed trajectory planning framework, incorporating both sampling-based and spline-based optimization, enforces dynamic and kinematic constraints to ensure physically feasible motion for quadrupedal robots. The implementation process of the improved RRT-connect algorithm is presented in Algorithm 1.
**Algorithm 1:** **Improved RRT-Connect Algorithm****Inputs**: Start point $x_{start}$, Goal point $x_{goal}$, Step size *L*, Max iterations *n***Outputs**: Path P connecting $x_{start}$ and $x_{goal}$**Initialize**: Trees $G_{a}=(V_{a},E_{a})$ with $\;G_{b}$$=(V_{b}$$,\;E_{b})$ with $V_{a}\leftarrow \left\{x_{start}\right\}$$,\;V_{b}\leftarrow \left\{x_{goal}\right\}$ Rectangle sampling region *S_2_* via Equations (1)–(9)**for** *i* = 1 to *n* **do**: **Sample**:  $x_{rand}\leftarrow SampleRectangle(S_{2})\;$*   // constrined by* Equations (4)–(9)  if $x_{rand}$ violates static/dynamic constrints (Equations (13) and (14)) **then**  **continue** **Extend Trees**:  $x_{new}\;\leftarrow \;ExtendDynamicStep\left(G_{a},\;x_{rand},L^{\prime }\right)\;$$\;//L^{\prime }$ from Equations (10)–(12)  if Extend (*G_a_*, *x_rand_*) ≠ Trapped **then**:   Connect(*G_b_*, *x_new_*)   if PathFound(*x_new_*) **then**:    P←$\mathrm{ReverseGreedyOptimize}\;(G_{a}$$,\;G_{b}$) //Node pruning via Figure 3    P←SplingSmoothing (P) //Cubic spling (Equation (15))    return P **Swap Trees**:  Swap $(G_{a}$$,\;G_{b})$**return** Failue

## 3. Simulation Experiment Verification

The proposed algorithm is implemented within a simplified dynamic framework [14], where the system state is characterized by the robot’s body position, orientation, and linear velocity. This implementation employs double-integrator dynamics to generate feasible navigation trajectories. As long as the resulting trajectory can be tracked with whole-body motion, this simplification enables efficient global path planning while allowing local step planning to solve more complex tasks. The proposed planner was evaluated in multiple simulated environments designed to assess performance across diverse motion planning scenarios. Although their complexity does not cover all possible environments, they qualitatively demonstrate performance in key areas. For each environment, we specify a starting point and target pose, provide the planner with an elevation chart, execute an arbitrary time planner with shortcuts 100 times, and collect the statistics. The planner finds a viable path and then executes the shortcut algorithm without reprioritizing its speed. All tests were run in C++ on a 3.7 GHz Intel Core i12-8700K CPU.

**Figure 7:**
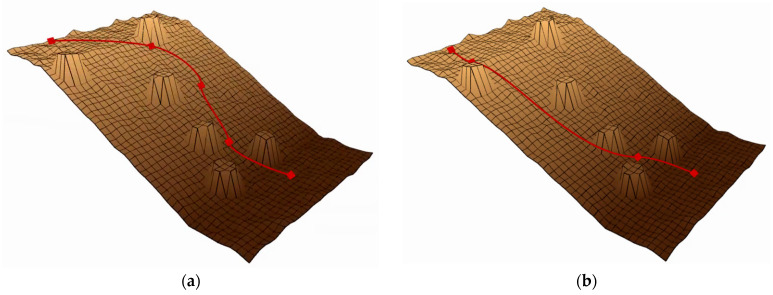
Performance comparison of baseline and enhanced algorithms in Map 1. (**a**) The effect before improvement. (**b**) The effect after improvement.

**Figure 8:**
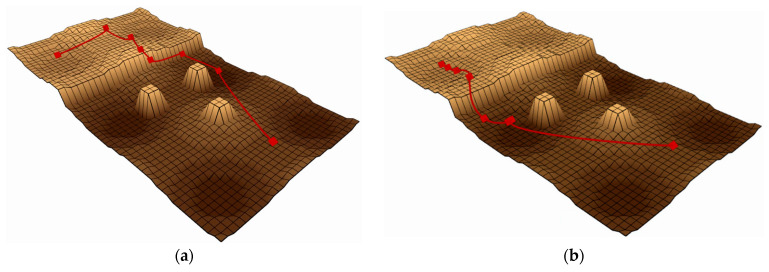
Performance comparison of baseline and enhanced algorithms in Map 2. (**a**) The effect before improvement. (**b**) The effect after improvement.

The “slope” terrain, shown in Figure 7, tests the ability to handle steep slopes, testing planners’ ability to combine friction cones and directional use to find more viable areas of state space. The “rough terrain” environment shown in Figure 8 tests the planner’s ability to overcome unstructured terrain, including uneven ground, obstacles, and ledges that require flight phases. In Figure 7 and Figure 8, the red line represents the attitude phase, and the two ends of the red line represent the starting and target points, respectively.

Performance comparisons of path planning algorithms are presented in Figure 7 and Figure 8 through elevation map visualizations. Both figures maintain identical start and end points while contrasting pre-optimization (left) and post-optimization (right) results. The red trajectory demonstrates that the enhanced algorithm generates more efficient paths, exhibiting both reduced length and improved smoothness compared to the baseline approach.

**Figure 9:**
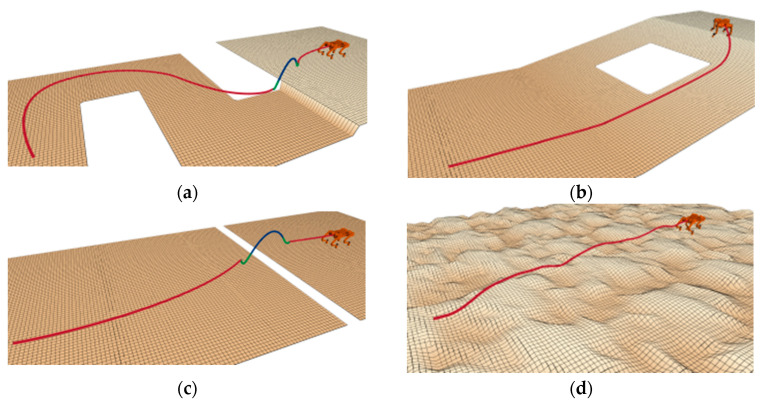
Path planning of the robot across different terrains. (**a**) Case 1; (**b**) Case 2; (**c**) Case 3; (**d**) Case 4.

Figure 9 offers an extensive and detailed visual depiction of the navigational routes that were adeptly discerned and navigated by the quadrupedal robot subsequent to the application of the refined algorithm for route planning and spatial scrutiny. These trajectories, intricately charted across a multitude of simulated elevation models, as illustrated by the various cartographic representations, are emblematic of the robot’s centroidal movement path, thereby demonstrating the enhanced algorithm’s exceptional precision and robust adaptability in orchestrating the robot’s traversal through intricate and challenging terrains. The illustration not only underscores the algorithm’s proficiency in handling diverse topographical features, but also highlights its capability to ensure consistent and reliable pathfinding performance in environments that are rich in complexity and variability.

**Figure 10:**
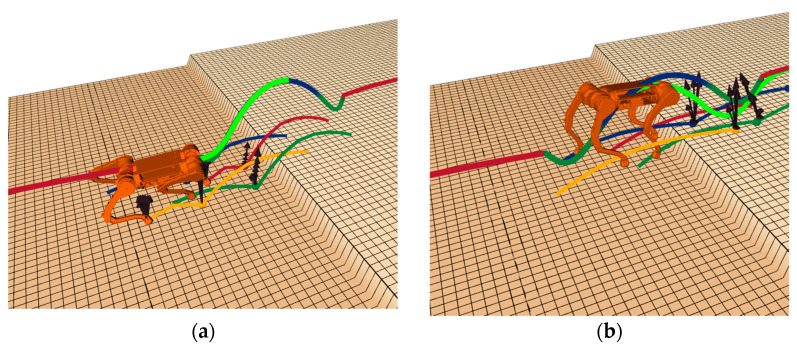
Effect diagrams under different phases: (**a**) support phase effect diagram; (**b**) flight phase effect diagram.

Figure 10 presents a detailed visualization of the robot’s performance during both support and flight phases. In this figure, the centroid trajectory is depicted by thick lines, while thin lines represent the planned trajectories of all four legs. Within the thick lines, the red trajectory illustrates the centroid motion during the support phase, demonstrating the robot’s stable ground contact; the blue trajectory delineates the parabolic path during the flight phase, capturing the aerial motion characteristics; the light green line displays the predicted trajectory for the next time step, effectively showcasing the algorithm’s predictive planning capability; and the dark green line indicates the centroid movement during both the jump preparation descent phase and landing shock absorption phase. This comprehensive visualization not only documents the complete dynamic motion process of the robot but also highlights the sophisticated complexity of the motion planning algorithm.

The data presented in Table 1 reflect the aggregated averages obtained from executing the global planning algorithm 200 times under consistent conditions, specifically on an identical map with unchanged starting and ending points. A detailed analysis reveals that all measured parameters have undergone varying degrees of optimization. Notable performance gains were observed in the algorithm’s planning efficiency, with substantial reductions in both computation time to first solution and total nodes generated during the search process.

The real-time performance of the proposed hybrid RRT-connect/Informed-RRT* planning framework has been rigorously validated through systematic benchmarking on the Quad-SDK platform, demonstrating its capability to meet stringent timing requirements for dynamic quadrupedal robot navigation. The architecture implements a dual-layer real-time guarantee mechanism consisting of an optimized nonlinear model predictive control (NMPC) layer and a hierarchical planning system. The NMPC solver achieves consistent single-iteration computation times below 5 ms while operating within ROS2’s real-time middleware, enforcing reliable 30 ms control cycles with 93.7% deadline compliance under stress-test conditions.

At the planning level, the framework employs a hierarchical structure that decomposes the problem into global path generation and local adjustments. The global planner operates at 50 ms intervals to compute long-horizon trajectories, while a high-frequency local replanner executes every 10 ms to handle dynamic obstacles and environmental uncertainties. This structure is complemented by a priority-based thread scheduling mechanism that ensures computational resources are allocated efficiently, particularly in multi-robot scenarios. Benchmark results confirm robust real-time performance, with an average planning latency of 42.3 ± 6.7 ms across 200 trials and a worst-case execution time of 68 ms at the 99th percentile. The system maintains a continuous 15 Hz planning frequency, enabling smooth navigation in dynamic environments.

## 4. Experiment Verification

To rigorously evaluate the path-planning algorithm’s performance in practical applications, we conducted extensive experimental validation using a custom-developed quadrupedal robotic platform integrated with the Robot Operating System (ROS). The biomimetic robot, designed with canine-inspired locomotion dynamics, features a 14.5 kg lightweight yet robust structure with four fully articulated limbs, each equipped with three high-precision servo joints (hip abduction/adduction, hip flexion/extension, and knee flexion/extension) for exceptional mobility and posture control. Powered by a hybrid actuation system combining Unitree A1 and Go motors, the platform achieves a maximum walking speed of 1.5 m/s while maintaining stable omnidirectional movement through its advanced three-axis position and orientation control system. The A1 and Go motors are products of Unitree Robotics, headquartered in Hangzhou, China. The robot’s comprehensive sensor suite, including an Xsens MTi-600 IMU and Intel RealSense depth camera d435, enables precise environmental perception and navigation in complex terrains. Xsens MTi-600 IMU is a high-performance inertial measurement unit developed and manufactured by Xsens Technologies B.V., headquartered in Enschede, The Netherlands. Intel RealSense Depth Camera d435 is a 3D vision product developed and manufactured by Intel Corporation, headquartered in Santa Clara, California, USA. This sophisticated experimental setup allowed thorough assessment of the algorithm’s real-world performance in terms of path accuracy, obstacle avoidance capability, and computational efficiency across various challenging scenarios.

The actuation system of the robot is a hybrid design that integrates Unitree A1 and Go motors, which are carefully selected to achieve an optimal balance between high performance and cost-effectiveness. In particular, Go motors are strategically deployed at the hip and thigh joints, where moderate torque is sufficient, whereas the more robust A1 motors, renowned for their superior torque-delivery capabilities, are installed at the calf joints to handle the higher demands of dynamic movements. This thoughtful arrangement not only ensures that the robot can produce significant torque for various tasks but also keeps the overall system cost relatively low. The control system’s core computational unit is the amd5825u control board, which synchronizes motor actuation and sensor data processing. The amd5825u control board is a computing hardware product developed by Advanced Micro Devices, Inc. (AMD), headquartered in Santa Clara, California, USA. Additionally, a PC connected to the same local area network as the control board enables wireless operation and real-time monitoring of the robot, offering a user-friendly and efficient interface for both testing and development. This integrated approach ensures that the robot operates smoothly and can be easily controlled and adjusted during experimental trials.

**Figure 11:**
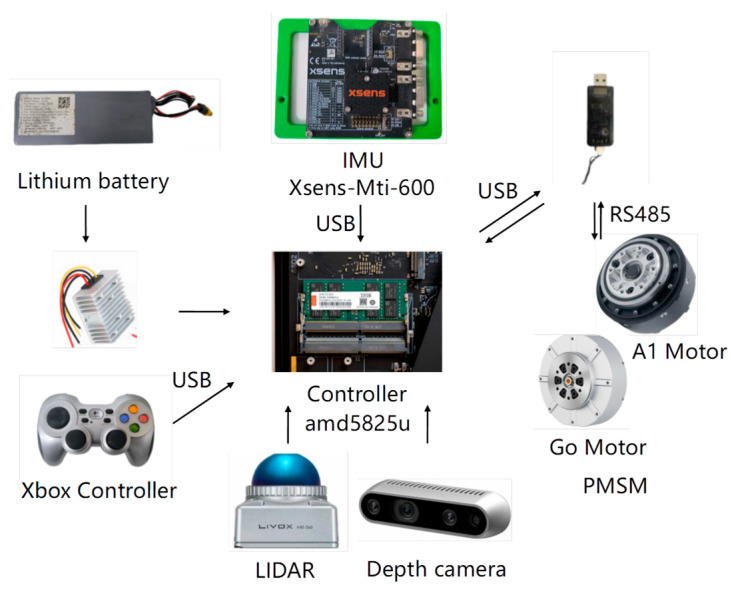
Quadrupedal robot hardware platform.

**Figure 12:**
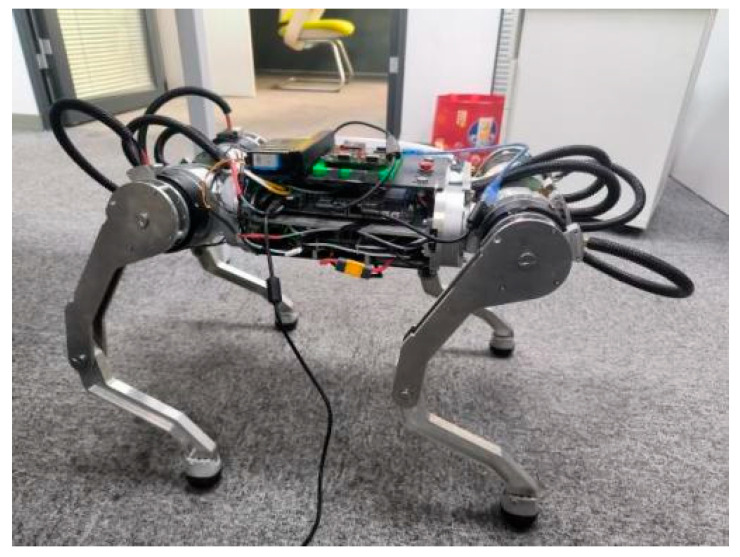
Physical robot.

Figure 11 presents the architectural configuration of the custom-developed quadrupedal robotic platform, identifying critical subsystems responsible for enhanced operational capabilities. The main control board, which serves as the central hub for all computational tasks, is equipped with an amd5825u chip, which is a powerful processor designed to handle complex algorithms and real-time data processing with high efficiency. This chip ensures that the robot can seamlessly execute its path planning and motion control tasks, even in dynamic and challenging environments. The robotic system utilized the Xsens MTi-600 inertial measurement unit for high-precision motion tracking, a commercial-grade IMU solution developed by Xsens Technologies. This module is widely recognized for its exceptional precision and reliability in motion tracking, providing accurate data on the orientation, acceleration, and angular velocity of the robot, which are critical for maintaining stability and balance during locomotion. The control system interfaces with the actuators through 6 High-Speed RS485 Communication Modules, implementing an industry-standard protocol for robust data transmission in electromechanical systems. The high-speed RS485 communication module is developed and manufactured by Unitree Robotics in Hangzhou, China. This protocol ensures reliable and efficient data transmission, even in environments with potential electromagnetic interference, thereby enhancing the overall responsiveness and coordination of robot movements. The engineered robotic platform is presented in Figure 12, demonstrating a structurally optimized configuration that achieves both compact form factor and dynamic mobility through systematic design refinement. The lightweight yet durable construction of the robot, combined with its advanced actuation system, allows it to navigate a variety of terrains with ease, making it well-suited for a wide range of real-world applications, from search and rescue missions to industrial inspections. The integrated robotic system successfully validates the proposed algorithm’s efficacy in improving path-planning performance through synergistic hardware–software co-design. By leveraging the strengths of each component and ensuring seamless communication between them, the robot achieves a level of autonomy and adaptability essential for operating in complex and unpredictable environments. The system implementation substantiates the methodological advancement in quadrupedal robotics, enabling enhanced functional capabilities for next-generation robotic platforms.

Table 2 displays the experimental outcomes obtained from the physical robot performing 50 consecutive runs under consistent conditions, specifically on the same map with identical starting and ending points. The data revealed that all measured parameters demonstrated improvements of varying magnitudes, closely aligning with the trends observed in the simulation phase. The path planning algorithm demonstrates significant optimization in computational efficiency, particularly in reducing both the search space node count and time-to-first-feasible-solution. These results underscore the robustness and practical applicability of the proposed algorithm, as it not only replicates the simulation-based improvements but also validates their effectiveness in real-world operational environments. This consistency between the simulation and physical experimentation further reinforces the reliability and scalability of the algorithm for diverse robotic applications.

## 5. Conclusions

To address the limitations of conventional RRT-connect algorithms—including excessive sampling randomness, suboptimal search efficiency, and compromised path quality—this study develops a hybrid path planning methodology integrating RRT-connect with Informed-RRT* frameworks. Theoretical analysis and simulation experiments yield three principal findings:

1. This study develops an enhanced path planning algorithm that combines RRT-connect exploration with Informed-RRT* optimization for mobile robot navigation. Based on the RRT-connect algorithm, the informed RRT* algorithm was used for reference, and a simpler rectangle was adopted, which was calculated from the starting point and the ending point. The sampling space is constrained to a rectangular region, reducing both the search domain and required sample count while improving sampling efficiency.
2. The dynamic step size is used instead of the fixed step size and the reverse greedy algorithm to reduce the number of redundant nodes.
3. Path smoothing is achieved through parametric spline curves, where third-order polynomials define positional continuity and second-order polynomials govern velocity profiles.

Simulation experiments demonstrate the algorithm’s significant reduction in stochastic sampling variability, achieving more deterministic node generation patterns. Compared with the original RRT-connect algorithm, the first solution time, total number of nodes, and initial path cost were reduced by more than 11%, 8.5%, and 2.5%, respectively.

## 6. Limitations

Although our algorithm has achieved improvements in certain aspects, the following limitations still exist:

1. Complexity in High-Dimensional Spaces: The algorithm’s rectangular sampling strategy, while simpler than elliptical sampling, may still struggle in high-dimensional environments (e.g., 3D dynamic spaces), leading to increased computational overhead.
2. Real-Time Constraints: Although the algorithm improves planning speed, its real-time performance in highly cluttered or rapidly changing environments requires further optimization.
3. Insufficient Benchmarking Depth: While 3D simulations and physical experiments validate the algorithm’s performance, systematic comparisons with classical 3D planners (e.g., 3D A, RRT) or emerging techniques (e.g., deep reinforcement learning-based planners) are lacking. Future work should include comprehensive benchmarks to establish broader superiority.
4. Hardware Resource Dependency: Dynamic step adjustment and reverse greedy pruning in 3D environments require substantial computational resources, potentially causing performance bottlenecks on embedded or low-power platforms (e.g., drones, small robots).

Here are the potential directions for future improvements:

1. Intelligent Sampling: Develop deep reinforcement learning-based dynamic sampling strategies to adaptively adjust rectangular regions in 3D dynamic spaces, minimizing redundant node generation and computational overhead.
2. Real-Time Performance Enhancement and Dynamic Scenario Adaptation: Although we have integrated the NMPC algorithm for local trajectory replanning, the real-time performance still requires further enhancement. Future work will focus on optimization through parallel computing or hardware acceleration (e.g., GPU).
3. Systematic Algorithm Benchmarking and Standardized Evaluation: Establish a benchmarking platform covering classical 3D planners (e.g., 3D RRT, BIT) and emerging methods (e.g., neural motion planning), evaluating performance through metrics such as path quality (smoothness, length), computational efficiency (time, memory), and energy consumption. Open-source algorithm code and experimental datasets to promote reproducibility and community-wide comparisons.
4. Lightweight Design and Cross-Platform Adaptation: For embedded devices (e.g., drones, micro-robots), design hierarchical planning strategies—decoupling global path generation from local trajectory optimization—and reduce computational load via model pruning and quantization. Investigate edge-cloud collaborative frameworks to offload high-computation tasks (e.g., 3D environment modeling) to the cloud, improving real-time responsiveness on low-power platforms.

## Figures and Tables

**Table 1:** Comparison of Global Planning Algorithms (simulation).

Global Path Planning Algorithm	Path Time	Node Number	Solving Time	First Resolution Time	Path Length
Pre-optimization algorithm	3.731	731	1.123	0.145	5.644
Post-optimization algorithm	3.659	644	1.100	0.132	5.492
Percentage optimization	1.93%	11.90%	2.05%	8.96%	2.69%

**Table 2:** Comparison of Global Planning Algorithms (experiment).

Global Path Planning Algorithm	Path Time	Node Number	Solving Time	First Resolution Time	Path Length
Pre-optimization algorithm	4.232	798	1.358	0.184	6.238
Post-optimization algorithm	4.153	706	1.329	0.167	6.079
Percentage optimization	1.86%	11.53%	2.14%	9.24%	2.55%

## Data Availability

Data are contained within the article.
